# Supplementary material for: Venomix: a simple bioinformatic pipeline for identifying and characterizing toxin gene candidates from transcriptomic data
Source: PeerJ. 2018 Jul 31;6:e5361. doi: 10.7717/peerj.5361 (PMC6074769; doi:10.7717/peerj.5361)

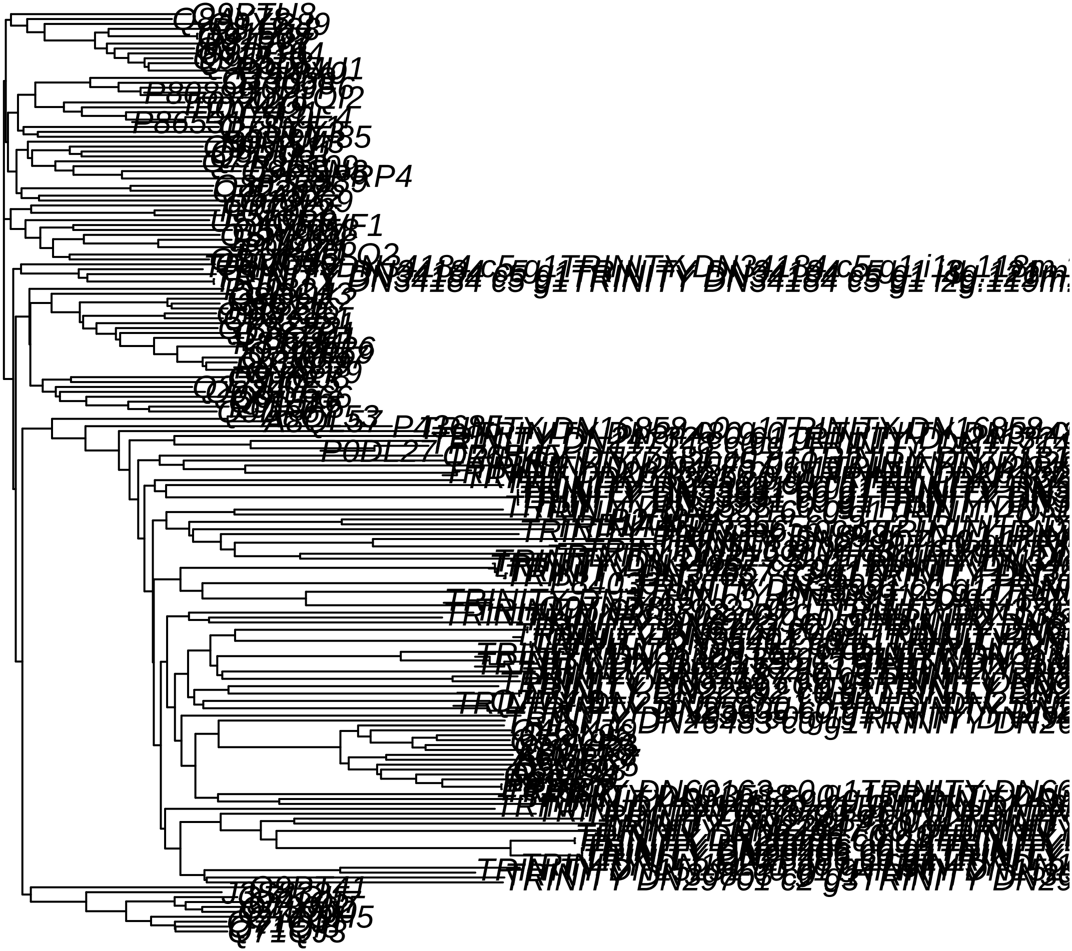

Supplement: Supplemental Information 3 [file peerj-06-5361-s003.gz › FinalOutput_E-6/Venom_prothrombin_activator_pseutarin-C_catalytic_subunit_12/finaltree.pdf]
